# Supplementary material for: Are carbon emissions trading and green financial instruments synergistic? -Comprehensive quantitative research based on content analysis
Source: PLoS One. 2024 Mar 7;19(3):e0298601. doi: 10.1371/journal.pone.0298601 (PMC10919601; doi:10.1371/journal.pone.0298601)
Supplement: S1 Data — (ZIP) [file pone.0298601.s001.zip › Data/地级市Z_F工作报告.zip!/║╙▒▒/╩»╝╥╫»╩╨/└┤╫╘╣1⁄2╓┌║┼í╛┬φ┐╦╩2╛▌═°í┐_2018.docx]

各位代表：

现在，我代表市人民政府，向大会报告工作，请予审议，并请各位市政协委员和列席人员提出意见。

一、2017年工作回顾

过去的一年，我们在习近平新时代中国特色社会主义思想的指引下，认真贯彻党中央、国务院的重大决策，全面落实省委、省政府的部署要求，在市委的坚强领导下，围绕建设现代省会、经济强市总目标，坚定不移贯彻新发展理念，坚定不移深化供给侧结构性改革，坚定不移践行以人民为中心的发展思想，经济建设呈现出稳中向好的发展态势，社会发展呈现出和谐稳定的良好局面。

一年来，我们坚持稳中求进，综合竞争力持续增强。全市生产总值完成6460.9亿元，增长7.3%，增速全省第一；一般公共预算收入完成460.7亿元，增长12.2%，总量全省第一；固定资产投资完成6310.1亿元，总量全省第一；规模以上工业利润增长8.2%，占全省的27.8%；社会消费品零售总额完成3296亿元，增长10.8%；城乡居民人均可支配收入分别达到32929元、13345元，均增长8.1%，高于经济增速；粮食生产稳定增长，实现“十四连丰”。全市常住人口城镇化率达到62.3%。成功创建全国文明城市和国家食品安全示范城市，实现了一年创“两城”，提升了全民文明素质和城市形象。

一年来，我们聚焦供给侧结构性改革，产业转型取得质的提升。产业结构调整成效明显，经济增长由主要依靠工业带动转为工业和服务业共同带动，发展动能由主要依靠传统产业转为战略性新兴产业、现代服务业和传统产业共同驱动，新势能正在积蓄，新动能加速发力。服务业增加值完成3066.4亿元，增长11.6%，占GDP的47.5%，对经济增长的贡献率达到75.1%，其中金融业增加值完成505.8亿元，增长17.7%，总量占全省的四分之一；规上高新技术产业增加值完成458.2亿元，占规上工业的19.4%，增长14.1%，高于规上工业增速10.5个百分点，其中生物医药、电子信息、高端装备制造业占高新技术产业增加值的84.8%；六大高耗能行业增加值增速下降5.8%，低于规上工业增速9.2个百分点；全年压减炼铁产能52万吨、火电产能19.9万千瓦，拆除、封停水泥产能523万吨，实现了新旧动能接续转换。大力推进农业现代化，“品牌农产品+直采直销”供给体系基本建立，现代农业园区达到280个，我市被评为全国农业农村信息化示范基地。

一年来，我们强力推进生态环境建设，大气质量明显改善。严格落实“1+19”大气治理政策体系，大力开展燃煤污染治理，完成气代煤42万户、电代煤1.8万户，淘汰燃煤锅炉5385台，主城区及周边三区一县实现散煤清零；完成1.9万家“散乱污”企业整治，852家企业基本完成挥发性有机物（VOCs）治理，6家重点企业实施搬迁；出台严管建筑施工扬尘“十二条”，685个在建工地全部落实“六个百分百”；停产整治矿山99个。切实加大环境监测监管力度，我市作为全国环保系统首个污染源和用电量“双在线”监控城市，被中央电视台宣传报道。全年空气质量优良天数达到151天，重度以上污染天数比上年减少20天，PM2.5平均浓度同比下降13.1%，下降率全省最高。特别是实施秋冬季攻坚行动以来，10月1日至12月31日，我市空气质量综合指数为7.55、下降48%，PM2.5平均浓度为85微克/立方米、下降54.8%，降幅在“2+26”城市中排名“双第一”，人民群众感受度明显好于往年，空气质量连续下降趋势实现逆转。同时，全面推进生态修复工程，高度重视水环境安全，积极推行河长制，大力实施“净土行动”，完成造林73万亩。

一年来，我们着力提标提质提档，城市建设实现新突破。按照“全域规划、多规合一”理念，启动城市总规修编。地铁1、3号线首开段正式运营，省会跨入“地铁时代”。石济客专通车运行，南绕城、平赞、西阜高速建设进展顺利，津石、石衡高速完成前期工作；启动中华大街南延等工程，打通裕华路等一批断头路，南二环西延、和平路高架西延等工程竣工通车。启动30平方公里重点区域海绵城市建设；18个综合管廊试点项目全部开工。1018辆纯电动公交车投入使用；获批北方地区冬季清洁取暖试点城市；成为首批国家装配式建筑示范市。滹沱河生态修复工程加快推进，龙泉湖、体育公园（北区）等公园基本建成，新建提升绿地990万平方米。全面开展环卫洁城、市容整治、非机动车停放管理等10个专项行动；取缔占道市场53处，拆除“双违”建筑面积685万平方米；完成主城区黑臭水体治理工作。开展交通秩序大整治，实施史上最严交规，道路通行效率明显提高，成为第三季度全国交通拥堵缓解程度最大的城市。县城攻坚行动成果丰硕，正定被评为全国文明县城，无极、深泽、赞皇成功创建省级园林县城，晋州、高邑获评省级洁净城市，鹿泉区铜冶镇入选全国第二批特色小城镇。城市吸引力进一步提升，人口净流入量逐年增长，我市成为吸纳北京外溢人口最多的城市。

一年来，我们着眼提高发展质量，创新能力显著增强。深度融入京津冀协同发展，抓住石保廊全面创新改革试验区建设机遇，重点推进153项改革措施，建成中关村天合科技成果转化服务、高新技术产品推广应用、传统产业智能改造和河北工业设计创新等4个平台；投入1160万元，重点支持与京津知名高校、科研院所合作项目15项；成立河北省军民两用技术交易等5个中心；新认定高新技术企业194家、科技型中小企业2912家，总数分别达到803家、9105家；设立研发机构的规上工业企业达到23%以上；科技进步贡献率达到56%。设立诺贝尔奖获得者工作站5家；院士工作站总数达到66家；吸引10名国家“千人计划”专家与我市开展创新合作。通过实施“人才绿卡”制度，引进高层次人才2900多名，人才高地雏形显现。

一年来，我们致力发挥市场的决定性作用，改革开放呈现崭新局面。持续深化“放管服”改革，在全省率先成立市县两级行政审批局，实现“二十六证合一”，行政许可事项办理时限平均减少15.5天，新增市场主体17.98万户。完成公共资源交易体制改革阶段性任务，实现六类资源进场交易。圆满完成“双随机、一公开”改革任务，在全省率先实现监管全覆盖。政府投资项目“代建制”改革顺利实施，全年代建项目23项。大力开展土地收储制度改革，推行城中村土地“整案制”收储，实行“先做土地、后做项目”，积极破解项目落地难问题；推进国有建设用地二级市场试点，科学划定永久性产业用地。扎实推进财税和投融资改革，设立蓝天产业转型、军民融合等产业基金，全市新增挂牌上市企业34家，新增贷款和存贷比创多年新高。开发区“四项改革”全面完成，园区承载功能不断增强。探索实施中俄“两国双园”合作模式，在新加坡、莫斯科成功举办专题招商会和优势产能推介会，石洽会、动博会、国际通用航空博览会知名度和影响力持续提升。全年实际利用外资13.9亿美元、增长14.2%，引进省外项目资金991亿元，外贸进出口总值和对外投资分别完成862.2亿元、15.8亿美元，占全省的25.5%和41.8%，对外开放进一步扩大。

一年来，我们积极培育内生动力，消费经济形成新亮点。积极适应中等收入群体不断壮大、消费结构加快升级、消费贡献不断提高的新形势，着力增强消费对经济增长的拉动作用，2017年社会消费品零售总额居全省首位，消费成为拉动经济增长的主要力量。充分挖掘市域丰富的旅游资源，成功举办首届旅发大会，与3省、8市88家景区实现旅游互联互通，叫响“红色西柏坡、幸福石家庄”旅游品牌，全域旅游格局初步形成，旅游业总收入达到962.6亿元，居全省首位。建成勒泰庄里街、万达室外步行街等13条特色商业街区，北国-勒泰、万达-怀特、新百-银座等城市商圈初具规模，省会夜经济消费渐成时尚，群众购物更加便捷；医疗保健、文化体育、教育培训、健康养老等服务性消费加快发展，共享式消费、体验式消费等成为新热点。全市手机用户1251.7万、光纤宽带用户274.6万、物联网用户219万，信息消费规模进一步扩大。

一年来，我们着力办实事惠民生，人民生活水平不断提高。持续加大民生投入，各级财政民生支出占公共预算支出的75.9%，老旧小区改造、居家养老服务、发放文化惠民卡等10件民生实事全部完成。精准推进脱贫攻坚，实现稳定脱贫6.5万人，196个村脱贫出列，平山县达到脱贫出列申报标准。不断提高保障水平，城镇新增就业18.26万人，农村转移劳动力5.86万人；企业退休人员养老金人均月增148.9元，城乡居民低保标准分别增长17%、18%；高度重视农民工工资支付问题，圆满完成治欠保支工作；房地产遗留问题涉及的39万户，已解决30.5万户，占总数的78.1%。统筹推进各项事业，创建普惠性幼儿园46所，主城区所有公办小学全部实行免费托管；市区23家公立医院全部取消药品加成，4家社区卫生服务中心入选全国百强，22个县（市）、区全部建成医养结合示范机构；10分钟健身圈不断完善，更新安装全民健身路径105条；创建全民阅读“书香石家庄”品牌，石家庄大剧院、丝弦剧院投入使用；正定开元寺考古取得重大进展，古中山国遗址列入国家考古遗址公园立项名单，行唐故郡东周遗址入选“2017年中国考古六大新发现”；平安省会建设全面加强，社会大局和谐稳定，近十年来首次实现全年未发生较大以上安全生产事故；“律师顾问、公证顾问”法律服务模式被评为2017年河北省十大法治成果。国防动员、双拥共建、优抚安置、民族宗教、外事侨务、人民防空、气象地震、妇女儿童、老龄、残疾人等事业都有了新的发展和进步。

同时，我们坚决贯彻全面从严治党的战略部署，严格落实中央八项规定精神，持之以恒纠正“四风”，切实加强政府系统党风廉政建设，为全市经济社会发展营造了良好环境。忠实履行宪法和法律赋予的职责，坚持依法行政，自觉接受市人大法律监督、市政协民主监督和社会舆论监督，办理人大代表建议415件，政协委员提案434件，按时办结率达到100%。

各位代表！过去的一年，我们坚持立足长远谋全局，注重对重大战略问题的谋划研究和顶层设计，积极推动和实施一系列思路创新、举措创新，在诸多矛盾交织叠加、多重困难挑战并存的形势下，顶住了压力，经受了考验，取得了来之不易的成绩。这是习近平新时代中国特色社会主义思想指引的结果，是省委、省政府关心支持的结果，是市委坚强领导的结果，也是全市上下共同努力的结果。在此，我代表市政府，向全市人民，向人大代表、政协委员，向各民主党派、工商联、无党派人士和人民团体，向驻石解放军、武警官兵和政法干警，向中直、省直驻石单位，向关心支持石家庄发展的国内外朋友，致以崇高的敬意和衷心的感谢！

在肯定成绩的同时，我们也清醒地看到我市发展不平衡不充分的一些突出问题。一是经济总量还不够大，占全省的比重不足五分之一，与全国先进省会城市的差距比较明显，尤其是县域经济总量偏小、占比偏低。二是发展质量和效益还不高，实体经济发展困难还不少，创新能力有待进一步提升，改革开放亟待突破。三是城市建设与现代省会定位不相适应，承载能力不强，特别是县城建设质量不高、服务功能不完善。四是生态环境治理任重道远，保护和修复任务艰巨，大气污染问题仍然突出。五是民生保障领域仍有短板，就业、教育、医疗、养老等方面还不能很好满足人民群众的需要。六是营商环境尚需改善，一些工作人员不担当不作为，服务质量和效率有待进一步提高。对此，我们一定坚持问题导向，以革故鼎新的勇气，努力把省会各项事业推向前进！

二、2018年目标任务和重点工作

今年是全面贯彻党的十九大精神的开局之年，是改革开放40周年，是决胜全面建成小康社会、实施“十三五”规划承上启下的关键一年，是加快建设现代省会、经济强市的攻坚突破之年。展望发展大势，我市正处于历史性窗口期和战略性机遇期，京津冀协同发展和雄安新区的规划建设，为我们在对接京津、服务京津中发展自己提供了有利条件；“一带一路”建设的加快推进，为我们提升开放型经济发展水平、增强经济发展新优势创造了难得机遇。特别是省委、省政府对省会工作高度重视，明确提出要举全省之力支持省会建设发展，为我们加快建设现代省会、经济强市提供了前所未有的强大动力。我们一定紧跟时代潮流，肩负起新时代赋予的历史使命，在先进城市体系中找标杆，从国家大战略中找动力，主动作为，勇闯难关，砥砺前行，努力创造出无愧于时代的新业绩。

今年政府工作的总体要求是：以习近平新时代中国特色社会主义思想为统领，全面贯彻党的十九大精神，认真落实省委九届六次全会、省委省政府对省会建设发展一系列指示要求，按照市委十届四次全会的部署，坚持稳中求进工作总基调，坚持新发展理念，紧扣我国社会主要矛盾变化，按照高质量发展的根本要求，统筹推进“五位一体”总体布局和协调推进“四个全面”战略布局，深化供给侧结构性改革，加快建设现代化经济体系，以“五抓五提升”为总抓手，统筹做好稳增长、促改革、调结构、治污染、惠民生、防风险各项工作，促进经济社会持续健康发展，加快推进省会现代化、国际化建设，打造区域中心城市，增强辐射带动能力，开创新时代现代省会、经济强市新局面。

经济社会发展的主要预期目标是：地区生产总值增长7.5%左右，一般公共预算收入、全部财政收入分别达到500亿元、1000亿元以上，实现“跨五越千”，服务业增加值增长11%左右，规上工业增加值增长5%左右，固定资产投资增长7%，社会消费品零售总额增长10%以上，城乡居民人均可支配收入增长8%左右，居民消费价格涨幅控制在3%左右，PM2.5平均浓度下降至74微克/立方米。

各位代表，进入新时代，我国社会主要矛盾已经转化为人民日益增长的美好生活需要和不平衡不充分的发展之间的矛盾，我国经济发展已由高速增长阶段转向高质量发展阶段。我们必须主动适应这一阶段性特征，立足我市经济社会发展现实需求和长远目标，坚持质量第一、效益优先，把高质量发展贯穿到各个领域，以高质量发展引领转型升级，以高质量发展赢得优势、赢得未来；必须把市委十届四次全会确定的“奋进新时代、担当新使命、激发新干劲、创造新业绩”作为鲜明主题，统一思想、凝聚共识，创新思路、完善举措，全力推动省会发展走在全省前列。集中力量做好以下八项重点工作：

（一）加快构建“4+4”产业发展格局，提升高质量发展水平。实现省会高质量发展，必须以高质量的产业为支撑。坚持把构建“4+4”产业发展格局作为主攻方向，尽快编制完善产业发展规划，壮大实体经济，提高产业竞争力，增强经济质量优势。

做强做优四大产业。一是新一代信息技术产业。重点推进北斗综合位置云服务平台等一批重大项目，建设国家卫星导航应用示范城市和研发制造基地；发展壮大微波、激光器和光电显示全链条产业，构筑光电显示产业集群；引进培育太赫兹、量子通信、物联网等前沿技术产业。全市信息技术产业增加值达到100亿元。二是生物医药健康产业。加快高端化制剂药、现代中药、基因工程药物、高性能医疗器械创新研发及产业化，提升制剂药占比；发挥好诺贝尔奖工作站的作用，促进国际生物医药尖端成果在我市转化；抓好石药新药制剂产业化、华药生物发酵基地等重大项目，创建新型生物医药技术国际创新试验区和国家康复器具产业园。全市生物医药健康产业增加值达到180亿元。三是先进装备制造业。依托中航华北，建设通航装备制造和运营中心；依托中车华北、翼辰等企业，建设全国轨道交通设施设备生产基地；依托中博、奇瑞等企业，建设华北地区新能源汽车制造基地，加速通用航空、轨道交通、新能源汽车等先进制造业集聚。全市先进装备制造业增加值突破100亿元。四是现代商贸物流业。推进全国现代商贸物流中心城市建设，抓好华润万象综合体、东南智汇城商业综合体等重点商贸项目；支持乐城·国际贸易城、塔坛国际商贸城等综合市场，积极承接北京批发业态疏解，建设内外贸一体化的大型批发交易市场；支持传统商业企业运用互联网技术创新经营模式，实现线上线下融合发展。做好物流空间布局规划，启动铁路物流港项目。加快国际展览中心建设，办好国际通用航空博览会、国际智能制造博览会等大型展会，全年举办大中型展会150场次以上。实施消费促进、电子商务进农村进社区、商贸物流配送体系建设、实体商业转型升级四大专项行动，持续释放消费潜力，增强消费拉动经济增长的基础性作用。

培育壮大四大产业。一是旅游业。加快全域旅游示范区建设，推动3A以上景区提档升级，谋划建设3-5条精品旅游线路，依托太行山高速开发旅游产业带；办好鹿泉、平山旅发大会，打造石家庄旅游品牌，全年旅游业总收入同比增长21%。二是金融业。大力发展开放式金融，加快金融创新开发区规划建设，以更优惠的政策、更高效的服务、更优良的环境，激发金融创新活力，集聚金融人才、机构和资产，培育区域性股权、期权、债权、产权市场，建设现代金融服务体系，增强金融产业实力，打造京津冀区域性金融中心；支持地方性金融机构加快发展，推进市级商业银行组建工作；加大挂牌上市企业培育力度，年内新增挂牌上市企业30家以上。全年金融业增加值增长10%以上。三是科技服务与文化创意产业。依托河北工业设计创新中心，扶持发展专业化公司，打造“设计+”产业链，建设全省最大的工业设计城，不断壮大软件开发、知识产权服务等科技服务产业规模。充分利用数字技术促进文化创意产业发展，加强数字动漫和游戏关键技术开发，带动动漫产业链条延伸拓展，打造以数字化产品、网络化传播、个性化服务为核心的文化创意产业集群。四是节能环保产业。重点聚焦京津冀和北方地区节能环保需求，大力发展环境监测检测和治理装备，加快大气污染、水污染、土壤污染防治设备的开发与产业化；支持发展循环利用和高效节能新型成套设备，推进资源循环利用和节能环保企业开拓国际国内市场；探索新型环保服务模式，引进培育一批综合环保服务企业。依托先河环保、河北科技大学研发优势，建设环保监测设备生产研发中心、制药工业污染控制工程技术中心，创建环保节能产业园区，做大节能环保产业规模。

大力推进军民深度融合。充分发挥河北军民两用技术交易中心作用，加强与涉军单位的战略合作，支持军民融合关键技术研发，构建“研发-交易-孵化-落地”的军民融合新模式，创建国家级军民融合创新示范区。推动中电科13所、54所等军工企业与市域企业深度合作，抓好通用航空产业基地、军民融合创新科技园、河北军鼎科技园等一批重点项目，加速军工技术民用化转移，打造军民融合产业集群。培育发展数字经济。数字技术作为关键生产要素，是经济结构优化和高质量发展的重要驱动力。加快数字技术、产品和服务在经济社会各行业中的应用，提高生产制造、消费流通、城市建设、社会治理、教育医疗等领域数字化、智能化水平，充分释放数字经济发展潜力。加快窄带物联网技术在计量抄表、智慧泊车、仓储物流、环境监测等领域的推广，建设“万物互联”石家庄；举办好国际数字经济博览会，打造全球数字经济交流合作平台。大力发展共享经济、现代供应链等新业态新模式，培育新的经济增长点。加快传统产业优化升级。深入开展“中国制造2025”和“互联网+”行动，落实《加快推进工业转型升级指导意见》，实施“互联网+制造业”融合发展试点示范工程，滚动推进100项重点技术改造项目，培育10个省级智能工厂（数字化车间）和2个智能制造示范园区。开展质量提升行动，支持企业、事业单位参与或主导国际、国家、行业标准的制定修订。鼓励企业增品种、提品质、创品牌，争创中国质量奖、中国工业大奖，年内中国驰名商标达到50件以上。坚持用市场化、法治化手段推进去产能工作，不折不扣完成省下达的压减任务。

（二）积极扩大优质投资，强化高质量发展支撑。优质高效投入是调整优化经济结构的重要基础。坚持投向、投量、投速、投效一起抓，多措并举、多点发力，增强经济高质量发展的内生动力和活力，争当经济建设排头兵。

集中力量推进项目建设。全面落实加快项目建设和扩大有效投资的“2+8”政策文件，继续实行“六个一”项目建设推进机制，开展项目集中开工和展评观摩活动，形成开工一批、建设一批、竣工一批、储备一批的滚动建设格局。今年计划实施市重点项目340项，其中续建项目120项、计划开工项目100项、前期项目120项，总投资6147亿元，年度计划投资723亿元。同时，进一步放宽民间资本进入领域，释放民间投资活力，促进民间投资快速增长。

强力实施精准招商。制定完善产业链招商指导目录，确定重点招商对象，采取小团组、专业化等方式，开展精准招商，引导外资投向战略性新兴产业、现代服务业等领域。精心组织石洽会、“招商引资月”等投资洽谈活动，加强与世界500强及国内外领军企业的对接合作，引进一批科技含量高、投资体量大的好项目。进一步强化党政“一把手”招商，今年每个县（市）、区引进3-5个对区域经济发展具有支撑作用的大项目。全年实际利用外资增长5%左右，引进内资增长10%。

提升开发区支撑作用。深化开发区改革，探索推行“产业引领、基金操盘”现代开发区发展模式，强化园区顶层设计，突出产业特色，明晰发展定位，提升开发区发展水平。对标国内先进开发区，进一步完善开发区基础设施，加快推进“新九通一平”建设。做好国家级、省级开发区对各类工业聚集区的托管工作，提高整体发展质量和管理水平。推进工信部、科技部重点支持的智能制造、中美科技创新等产业园建设，打造面向未来的产业集群。全市省级以上开发区主营业务收入增长20%以上。

破解项目用地制约。按照“补改结合”的要求，大力推进高标准农田建设，加快实施补充耕地项目，增加占补平衡指标储备。坚定不移推行“先做土地、后做项目”，重点做好“整案制”、大片区土地收储，为项目落地、产业转型提供要素保障。

（三）大力实施创新驱动战略，增强高质量发展动力。创新是引领发展的第一动力，是建设现代化经济体系的战略支撑。坚持创新竞进，加快构建以科技创新为核心，多领域互动、多要素联动的综合创新生态体系，推动经济发展质量变革、效率变革、动力变革。

大力培育创新主体。实施高新技术企业、科技型中小企业“双倍增”计划，打造产业链配套的创新型产业集群，年内新增高新技术企业150家、科技型中小企业1300家。发挥企业科技创新的主体作用，加快规模以上工业企业研发机构建设，引导全社会加大研发投入力度，年内研发经费支出占生产总值比重提高到2%。组织开展国家知识产权优势企业、示范企业培育工作，遴选25家企业开展知识产权管理标准认证，万人发明专利拥有量达到5.6件。

加强创新平台建设。聚焦提升创新能力，发挥“四个平台”作用，解决技术成果转化难、推广应用难、融资贷款难等问题。大力支持国家级、省级重大创新平台建设，提升关键共性技术研发能力，促进相关产业向高端化发展。鼓励我市企业联合高等院校、研发机构成立产业技术研究院，推动企业、科研单位合作共建产学研联盟，争取国家重点实验室、重点基础研究项目落户我市，年内新增市级以上创新平台30家。实施孵化器和众创空间倍增计划，新增市级以上孵化器4家、众创空间15家以上。

引进培养创新人才。加大“人才绿卡”政策落实力度，引进一批尖端创新人才和高层次创新团队。支持高等职业教育提升规模水平，鼓励企业与专业院校合作，联合培养我市主导产业急需的专业技术人才。尊重支持企业家，营造企业家成长环境，激发和保护企业家精神。全年新增院士工作站5-8家，认定科技领军人物15名、科技创新团队15个。通过厚植人才优势，让各类人才创造力竞相迸发，让创新成为经济发展的不竭动力！

（四）深化改革扩大开放，激发高质量发展活力。改革是活力之源，开放是高质量发展的必由之路。认真落实中央和省委省政府的总体部署，确保改革举措落地生效，推动形成全方位开放新格局，当好改革开放领头羊。

全面释放改革红利。充分发挥改革对高质量发展的先导性作用，持续推进增添发展活力的改革措施。一是深化“放管服”改革。在“多证合一”的基础上，实现“四十证合一”。扩大政府投资项目“代建制”改革范围，提高政府投资效益。二是深化财政和金融改革。科学划分市以下财政事权和支出责任，推进绩效预算管理改革。统筹整合政府资产资源，组建市政府直属金融控股集团，加强与金融机构密切合作，最大限度支持地方经济社会发展。完善贷款担保、风险补偿等机制，鼓励金融机构创新金融产品、畅通信贷渠道，通过投贷联动、银保合作、融资租赁、知识产权质押等形式，加大对科技创新、环保治理、旅游开发、住房租赁、棚户区改造等领域的支持力度。整合政府政务信息、企业信用信息、金融产品信息，建立金融服务大数据平台，支持“银政通”“云税贷”“按居贷”等银行信贷平台做大做强，促进金融和实体经济的良性循环，有效解决企业融资难和贷款风险防控问题。三是深化科技体制改革。深入推进石保廊全面创新改革试验区建设，全面深化科技计划管理、科技投入机制、科技成果转化收益分配等改革，加强知识产权创造、运用、保护。四是深化国企国资改革。完善国有资产监管体制，年内完成市级经营性国有资产集中统一监管；改革国有资本授权经营体制，组建国有投资运营公司，提高国有资本配置效率。五是深化住房制度改革。加快建立多主体供给、多渠道保障、租购并举的住房保障制度，综合施策稳控房价，严禁出现地王现象，继续解决历史遗留问题，确保房地产市场健康发展。

推动更高层次对外开放。树立全球视野和大开放观，拓展开放的范围和层次，加速融入“一带一路”，培育开放型经济新优势。一是大力发展“陆港经济”。依托正定国际机场、综合保税区，建设铁路、航空、公路“三港合一”的国际综合物流基地，推动物流基地与产业园区联动发展；配套设置铁路口岸，申报设立正定海关和进境商品指定口岸，打造全口岸型“无水港”城市；筹备开通石家庄中欧班列，打通直达欧洲、中亚的国际贸易物流大通道。二是加强国际产能和科技合作。加速推进“两国双园”建设，搭建海外高新技术转化平台、物流仓储平台和生产加工基地，重点抓好辰邦集团匈牙利海外仓、迈瑞公司波兰农产品深加工等项目建设，拓展对外交流合作的渠道和领域，推动优势企业“走出去”，加快发达国家科技成果“引进来”。三是推进贸易强市建设。加大对重点出口企业的扶持力度，培育一批高技术、高附加值出口企业。年内建设10个以上县域特色产业外贸基地。推动石家庄综合保税区跨境电商产业园、365网络科技集团跨境电商平台建设，布局建设海外仓和名优商品展销中心。全市外贸进出口总值增长8%左右。

全面融入京津冀协同发展。建立健全常态化的沟通协调和开放合作机制，以交通、产业、生态三大领域协同发展为重点，加强与京津的互联互通，推进京石城际铁路前期工作，加快津石高速、石衡高速建设，力争南绕城、平赞、西阜高速公路建成通车；积极承接北京非首都功能疏解和产业转移，着力吸引天津的企业向我市延伸产业链条，建立专业性“飞地园区”，推动正定科技新城、高新区高端生物医药产业基地、栾城国际智能制造协同创新中心等平台建设，促进京津科技成果转化项目落户我市。加强与雄安新区的沟通联系，紧盯雄安新区的产业布局和政策导向，调整完善我市发展思路和重点，实现与雄安新区产业上下衔接、错位发展。

（五）提升城市品位和影响力，加快建设现代省会。城市是吸引聚集产业、促进高质量发展的重要载体。围绕现代化、国际化城市标准，搞好顶层设计，优化空间布局，完善承载功能，强化综合治理，打造区域中心城市，增强辐射带动能力。

实施空间拓展工程。对标北京、雄安新区规划设计先进理念，按照“多规合一”要求和“内疏外聚”思路，高标准完成新一轮城市总体规划编制。制定四组团县区与中心城区一体化发展实施意见，加快推进都市区一体化发展。统筹推进滹沱河南岸北岸协调互动发展，推动调整滹沱河地下水水源地保护区和城区段南岸高标准行洪区范围，释放主城区发展空间。加快正定县（正定新区）建设步伐，国际展览中心、图书馆、规划馆、青少年宫全部投用，年底前4平方公里先导区道路全部通车，水电气暖讯全部配套。继续抓好正定古城建设重点工程，切实保护传承古城历史文化价值。

实施道路畅通工程。推进绕城公路前期工作，重点实施省道339、337改造项目，力争年底前开工建设。启动307国道、新元高速城市化改造以及东二环南延、建华大街南延、和平路高架东延等工程前期工作。基本完成联盟路-石纺路-丰收路连贯工程。实施解放大街及中央绿轴建设，完成东半幅征地拆迁和槐安路以南西半幅建设。强力推进公交都市建设，加快构建“1公里步行、3公里自行车、5公里公交、长距离轨道为主”的一体化公共交通体系，创建全国公交都市示范市。

实施功能提升工程。加快中央商务区建设，对车辆厂、解放广场、老火车站、长途客运站、湾里庙、南三条等片区，进行高标准规划和综合整治，打造城市记忆与现代产业交融、彰显省会特色的标志性区域。加快地铁1、3号线二期、3号线一期两边段、2号线一期工程建设，积极跑办4、5、6号线建设规划批复。抓好保障性安居工程建设，确保完成棚户区改造、公租房建设任务。推广装配式、被动式建筑，全面开展被动房试点工作。启动棉一旧厂区、华药旧厂区改造，建设科创文化园。抓好地下综合管廊项目，有序推进国家综合管廊试点城市建设。新建提升主城区14条供水主管道。对公里街、站前街等10条道路进行雨污分流改造。实施主城区30平方公里重点区域海绵城市建设。10月1日前，完成滹沱河生态修复工程藁城段河槽治理项目。启动环城水系整体改造提升工程，建设民心河西线、太平河东段景观绿道。建成铁路文化公园、西环公园等6座公园，对主要街道园林绿化进行艺术性改造，开展立体绿化和垂直绿化，提升城市园林绿化品质。

实施治理提质工程。发挥省会资源优势，支持移动、电信、联通等通信网络运营商，加快IPv6、5G商用进程，推动大数据、云计算、物联网、人工智能在城市治理、政务服务、智能制造、电子商务等领域的应用，加速推进智慧城市建设。提升城市净化亮化美化水平，深度实施“以克论净”保洁模式，主街主路水洗机扫全覆盖；建设桥西区、裕华区、高新区大型生活垃圾压缩站，谋划建设1座建筑垃圾处置场；取缔二环内全天候占道经营市场；完成主城区停车场规划，力争建成10个停车场；实施电线入地工程，整治城市街道外立面，规范牌匾广告，对中山路（中华大街-建设大街）3公里街区进行整体景观提升；对城市周边铁路、公路沿线重点区域开展整治行动，严格管控用地、建设行为。巩固拓展和提升全国文明城市创建成果，精准对标国家卫生城市标准，全力以赴创建国家卫生城市，让石家庄成为宜居之城、美丽之城、希望之城！

实施县城建设攻坚工程。以创建“园林城、卫生城、文明城、洁净城”为抓手，强化督导考评，争取更多县城进入全省前列。拆除违章建筑，年底前实现“双违”清零；增强承载功能，集中供热和清洁能源供暖率达到80%，县级市和县城污水处理率分别达到94%和88%以上，生活垃圾无害化处理率分别达到95%和93%以上；建设精品工程，每个县（市）累计完成24公里以上绿道绿廊建设，新建改建至少1座四星级以上精品公园，高标准打造1条迎宾景观大道、2条标志性街道、3条城市景观路。年内所有县建成省级园林县城，排名全省后30名的县全部出列，力争建成1-2个国家级特色小城镇。

（六）强力实施铁腕治污，推动生态环境持续好转。绿色是高质量发展的普遍形态，高质量是绿色发展的内在属性。自觉践行绿水青山就是金山银山的理念，坚持生态优先、绿色发展，在治理污染、修复生态中加快营造良好人居环境。

突出大气污染治理。瞄准率先退出全国重点城市空气质量“倒十”目标，全民共治、源头防治，坚决打赢蓝天保卫战。加大散煤治理力度，继续实施清洁能源替代，最大限度提高农村清洁采暖比例；控制工业燃煤总量，年底前全部淘汰全市范围35蒸吨/小时及以下燃煤锅炉，燃煤锅炉全部安装在线监测，稳妥压减电煤，全市煤炭消耗量净减400万吨；彻底整治“散乱污”企业，加快高耗能企业改造升级步伐，完成10家企业搬迁。禁煤区决不允许散煤复燃，已关闭的“散乱污”企业决不允许复产，生产低端劣质产品的企业决不允许在石家庄立足。加强扬尘治理，按照“六个百分百”要求，对工地、渣土运输车辆实施24小时全程管控。对“三区两线”主体灭失的12个露天矿山进行修复绿化。加强机动车尾气治理，加大对重型柴油车的排放检测力度，禁止国一、国二标准车辆进入主城区；加大充电桩建设力度，推广使用新能源车。

深化水和土壤污染防治。深入实施“水十条”，全面落实河长制，加强水质预警监测系统建设，强化水源地保护和入河排污口管理。加强黑臭水体排查，对新发现的黑臭水体年底前完成整治。加强地下水超采治理，全面开展湿地恢复建设，促进全市水生态总体改善。全面实施“土十条”，突出重点区域和行业，强化污染废弃物全流程管控，防治农村面源污染，开展土壤修复治理，促进土壤资源永续利用。

推进生态绿化建设。实施国土绿化三年行动计划，落实京津冀生态环境支撑区规划，坚持林果并重、增林扩绿与管理增效相结合，规划建设一批市级、县级森林公园；在沿河、沿路、沿城和镇旁、村旁开展成片造林绿化，全年完成造林70万亩。

强化生态环境监管。严格执行“问题、责任、整改、效果”四个清单制度，完善市县乡村四级信息网络监管体系，实施全市乡镇空气质量大排名，切实把压力传导到基层。强化对重污染天气的应对和处置，完善大气环境观测、大气污染监管等智慧环保平台建设，对重点污染源实施24小时监控，对恶意违法排污行为“零容忍”，实行顶格处理，保持铁腕治污的高压态势，以最严肃的态度、最严格的标准、最严厉的措施，坚决打赢污染防治攻坚战！

（七）落实乡村振兴战略，推进农业农村现代化。坚持农业农村优先发展，按照产业兴旺、生态宜居、乡风文明、治理有效、生活富裕的总要求，统筹谋划各项建设，打通城乡产业融合、生态环境融合、公共服务融合之路，推进乡村全面振兴。

推动农业全面升级。乡村振兴，产业兴旺是重点。调整优化农业生产布局，加快打造“一圈两区”现代农业发展格局。落实农业供给侧结构性改革三年行动计划，在稳定粮食、畜禽、蔬菜、果品生产的基础上，推进农业由增产导向向提质导向转变。实施“农业质量提升年”行动和品牌培育计划，年内市级以上现代农业园区、示范家庭农场和农民合作社实现标准化生产全覆盖，品牌农产品实现二维码质量可追溯，创建一批农产品质量安全县（市）；推进“三品一标”认证，争创省级特色农产品优势区和区域公用品牌。开展农业生产“三节”行动，推动生产绿色化、生态化。支持发展智慧农业、“互联网+农业”，年内实现电子商务综合服务平台和电商销售网点全覆盖。大力培育田园综合体等现代农业新模式，创建6-8个市级美丽乡村旅游度假区，推动农村一二三产业融合发展。

推动农村全面进步。乡村振兴，改善环境是关键。按照抓重点、补短板、强弱项的要求，推进城乡公共服务均等化，实现要素自由流动、平等交换。认真落实农村人居环境整治三年行动计划，以垃圾污水治理、“厕所革命”“四好农村路”、村容村貌整治为重点，改善农村生产生活条件。实施数字乡村战略，开发适应“三农”特点的信息技术、产品应用和服务。开展平安乡村创建行动，创新乡村治理体系。深化农村重点领域改革，整合财政涉农资金，撬动社会、金融和产业资本支持农村发展；全面完成承包地确权登记颁证工作，开展农村集体资产清产核资、集体成员身份确认，推进承包地经营权依法融资担保、入股从事农业产业化经营，推动资源变资产、资金变股金、农民变股东。年内农村集体产权制度改革村数达到1000个以上。

推动农民全面发展。乡村振兴，生活富裕是根本。实施强村富民行动，大力培育家庭农场、合作社、龙头企业和农业产业化联合体，发展多种形式适度规模经营。年内培育市级示范家庭农场和农民合作社各100家，建成县级以上农业产业化联合体100家以上。促进小农户和现代农业发展有机衔接，扶持小农户发展生态农业、设施农业、体验农业、定制农业，拓展增收空间。实施新型职业农民培育工程，大规模开展职业技能培训，支持鼓励更多年轻人务农兴农。开展乡村就业创业行动，促进农村劳动力转移就业，多渠道增加农民收入。建立有效激励机制，鼓励吸引社会各界和各类人才投身乡村建设。加强农村科普工作，提高农民科学文化素质。开展移风易俗行动，提升农民精神风貌，培育文明乡风、良好家风、淳朴民风。

实施精准脱贫攻坚。乡村振兴，摆脱贫困是前提。坚持把提高脱贫质量放在首位，确保年内实现200个村、5万贫困人口稳定脱贫，1-2个贫困县达到脱贫出列标准。继续推进“七个一批”专项行动和基础设施建设“七大工程”，大力发展脱贫产业，建立“贫困户-农民合作社-龙头企业-电商平台”的利益联接机制，拓宽贫困户增收渠道。继续实施深度贫困村“五包一”定点帮扶，确保特困群体稳定脱贫。扎实推进异地扶贫搬迁，推动集中安置区与脱贫产业区“两区同建”。对丧失劳动能力的特殊贫困人口，确保病有所医、残有所助、生活有兜底。深入开展扶贫领域腐败和作风问题专项治理，切实加强扶贫资金管理，强化脱贫攻坚责任和监督，坚决打好精准脱贫攻坚战，让贫困人口尽快搭上小康和富裕的快车！

（八）倾心尽力保障改善民生，增进群众获得感、幸福感、安全感。共享是高质量发展的根本目的。始终把人民利益摆在至高无上的地位，紧扣人民群众日益增长的美好生活需要，让改革发展成果更多更公平惠及全市人民。

优先发展教育事业。抓好普惠性学前教育，解决中小学生课外负担重、“择校热”“大班额”等问题。推动城乡教育均衡发展，推进县域内城乡义务教育一体化改革。大力发展现代职业教育，年内职教园区4所学校实现搬迁入驻。推进高等教育内涵式发展，鼓励高等院校争创“双一流”。继续实施山区教育扶贫工程，提升教育教学整体水平。加强京津冀教育交流合作，加大校长教师培养力度，提高教师队伍整体水平，让家门口的好学校好老师多起来。

提高就业质量和人民收入水平。落实创业担保贷款政策，出台就业补助资金管理办法，加快创业孵化基地建设。做好高校毕业生、农村转移劳动力、建档立卡贫困户、去产能企业职工等重点群体就业工作。实施技能人才等七大群体激励计划，带动城乡居民增收。全年城镇新增就业11.3万人，城镇登记失业率控制在4%以内，农村劳动力转移就业5.15万人。

加强社会保障体系建设。进一步完善养老保险省级统筹制度改革，做好退役军人养老保险关系接续工作。全面推行以按病种付费为主的多元复合式医保支付方式改革，城乡居民基本医保财政补助标准再提高40元。加快老龄事业发展，探索开展长期照护险试点。提升社会救助、优抚安置能力，关怀残疾人等弱势群体。

推进健康石家庄建设。抓好医联体建设和分级诊疗，加强家庭医生签约服务；全面落实药品采购“两票制”，严格控制医疗费用不合理增长。推进市一院赵卜口新院区建设，启动市妇幼保健院项目，市四院谈固新院区、市中医院东院区建成投用。加强基层医疗卫生设施和人才队伍建设，创建20个国家级“群众满意乡镇卫生院”。深化国家中医药综合改革试验市建设，创建全国基层中医药工作先进市。继续做好人口和优生优育工作。推进医养结合型养老示范基地建设，年内打造3-5个医养结合精品工程。

繁荣发展文化和体育事业。建立健全市县乡村公共文化服务设施网络，深入实施文化惠民工程。开工建设市博物馆和档案馆新馆，扩大文化惠民卡发放范围。推出更多具有石家庄特色的新时代文艺精品力作，办好第二届全国梆子声腔优秀剧目展演活动。建设中山古城考古遗址公园，做好正定开元寺、行唐故郡东周遗址考古和保护。因地制宜建设一批群众身边的休闲娱乐设施，新建改建射击馆、全民健身中心、中山体育馆等大型场馆，确保河北省第十五届运动会、第九届残疾人运动会圆满举行。

加强和创新社会治理。推进更高质量的“平安省会”建设，严格落实社会治安综合治理责任制，构建公共安全防控网络。大力开展扫黑除恶专项斗争，打击震慑黑恶势力犯罪，铲除黑恶势力滋生土壤。建立新型矛盾排查化解常态化机制，依法规范信访秩序，深入推进信访积案大化解“双百日”攻坚清仓行动，及时有效解决群众合理诉求。严厉打击非法集资、非法证券活动，有效防控金融风险。毫不放松抓好安全生产，建立安全监管责任清单，持续开展安全隐患大排查、大整治，坚决防范重特大生产安全事故。高度重视食品药品安全，巩固“国家食品安全示范城市”创建成果，全力维护人民群众生命健康安全。

加强全民国防教育、国防动员和后备力量建设，做好军队退役人员安置和服务工作，着力解决随军家属就业和子女入学问题。充分发挥工会、共青团、妇联等人民团体桥梁纽带作用。继续做好民族宗教、妇女儿童、残疾人、外事侨务、人防、档案、气象、防震等各项工作，促进各项社会事业全面进步。

继续办好利民惠民十件实事：一是对主城区100个老旧小区内的道路、绿化、安全、排水等基础设施进行改造。二是建成滹沱河城区段19公里生态景观长廊，免费向市民开放。三是对主城区49座公园（广场）进行无障碍改造，新增街旁绿地15万平方米。四是打通中华大街南延等10条断头路，为市民出行提供方便。五是在主城区16个路段设置可变车道、潮汐车道，在槐安路20个路口设置二次过街人行横道灯。六是为基层社区设置100个首席专家工作室、培养100名首席高血压医师和100名首席糖尿病医师，选拔100名高级医学专家每周到基层出诊不少于1天。七是建立新生儿出生缺陷防治机制，落实三级预防措施，新生儿疾病免费筛查病种增加到5种。八是新创建30所普惠性幼儿园。九是在社区安装全民健身路径100条，在农村实施农民体育健身工程100个。十是在主城区新建标准化公厕100座。

三、努力建设人民满意的政府

适应新时代、聚焦新目标、落实新部署，必须始终心系民生、肩扛责任、脚踏实地，在敢为上勇担当，在能为上增才干，在有为上重实效，努力打造廉洁、务实、高效、法治的政府。

（一）从严治政，保持清正廉洁本色。深入宣传贯彻落实党的十九大精神，旗帜鲜明讲政治，严守政治纪律和政治规矩，强化“四个意识”，坚定“四个自信”，始终同以习近平同志为核心的党中央保持高度一致，把习近平新时代中国特色社会主义思想贯穿到政府工作的全过程，转化为推动省会发展的生动实践。牢牢扭住主体责任“牛鼻子”，强化压力传导，紧盯重点领域、重点部门，严肃查处违纪违法行为和问题；整治发生在群众身边的“微腐败”，坚决纠正损害群众利益的行为，以反腐倡廉的实效取信于民。

（二）牢记使命，严格履行政府职责。扎实开展“不忘初心、牢记使命”主题教育，坚定理想信念，坚定思想自觉、政治自觉、行动自觉，以时不我待、只争朝夕的精神状态，高标准履行政府工作职责，努力为全市人民创造更加美好的生活。坚持大视野、高站位，强化省会担当，不断破解经济社会发展中的瓶颈和制约，努力在新时代有新作为。坚持运用战略思维、创新思维、辩证思维、法治思维、底线思维谋划工作，抓重点、抓难点、抓关键，全力推动政府各项工作实现新突破。

（三）勇于担当，提升政府执政能力。围绕适应新时代、新要求，加强学习和实践，不断提高把握和推动高质量发展的能力和本领。牢固树立“幸福都是奋斗出来的”理念，以钉钉子精神做实做细做好各项工作，下定决心打好攻坚硬仗，以更强烈的责任担当确保各项决策部署落地见效。充分凝聚干事创业的正能量，支持干事者、保护改革者、鼓励担当者，以实干推动发展，以实干赢得未来。

（四）依法行政，加快建设法治政府。严格按照法定权限和程序履行政府职能，确保政府一切工作在法律的框架下进行。健全重大事项集体决策机制，提高制定公共政策公众参与度。自觉接受市人大的法律监督、市政协的民主监督和社会公众的舆论监督。认真办理人大代表建议和政协委员提案，主动接受市人大执法检查和专题质询，落实重大事项向市人大报告和向市政协通报制度。严格规范公正文明执法，加大关系人民群众利益等重点领域的执法力度。进一步拓宽政务公开渠道，增强政府工作的透明度和公信力。

（五）转变作风，营造良好发展环境。严格落实中央八项规定和实施细则精神，深入开展纠正“四风”和作风纪律专项整治，持续把作风建设和优化发展环境引向深入。大兴调查研究之风，深化“双问计”活动，找准短板弱项，解决实际问题。进一步优化营商环境，开展“双创双服”活动，重拳治理“三乱”问题。强化督查，狠抓落实，对各项重点工作严格考核问责，以更实的作风、更严的要求、更高的标准，推动各项事业发展迈上新台阶。

各位代表！责任呼唤担当，使命引领未来。让我们紧密地团结在以习近平同志为核心的党中央周围，在省委、省政府和市委的坚强领导下，高举习近平新时代中国特色社会主义思想伟大旗帜，忠诚履职、埋头苦干、拼搏奋进，为决胜全面建成小康社会，加快建设新时代现代省会、经济强市而不懈奋斗！
